# Determination of the best knot and bandwidth in geographically weighted truncated spline nonparametric regression using generalized cross validation

**DOI:** 10.1016/j.mex.2022.101994

**Published:** 2023-01-04

**Authors:** Robiansyah Putra, Muhammad Ghani Fadhlurrahman, Gunardi

**Affiliations:** Department of Mathematics, Faculty of Mathematics and Science, Universitas Gadjah Mada, Yogyakarta, Indonesia

**Keywords:** Spatial regression, Nonparametric regression, Morbidity rate, Kernel function, Geographically Weighted Truncated Spline Nonparametric Regression (GWTSNR)

## Abstract

This study proposes the development of nonparametric regression for data containing spatial heterogeneity with local parameter estimates for each observation location. GWTSNR combines Truncated Spline Nonparametric Regression (TSNR) and Geographically Weighted Regression (GWR). So it is necessary to determine the optimum knot point from TSNR and determine the best geographic weighting (bandwidth) from GWR by deciding the best knot point and bandwidth using Generalized Cross Validation (GCV). The case study analyzed the Morbidity Rate in North Sumatra in 2020. This study will estimate the model using knot points 1, 2, and 3 and geographic weighting of the Kernel Function, Gaussian, Bisquare, Tricube, and Exponential. Based on data analysis, we obtained that the best model for Morbidity Rate data in North Sumatra 2020 based on the minimum GCV value is the model using knots 1 and the Kernel Function of Bisquare. Based on the GWTSNR model, the significant predictors in each district/city were grouped into eight groups. Furthermore, the GWTSNR is better at modeling morbidity rates in North Sumatra 2020 by obtaining adjusted R-square = 96.235 than the TSNR by obtaining adjusted R-squared = 70.159. Some of the highlights of the proposed approach are:

• The method combines nonparametric and spatial regression in determining morbidity rate modeling.
• There were three-knot points tested in the truncated spline nonparametric regression and four geographic weightings in the spatial regression and then to determine the best knot and bandwidth using Generalized Cross Validation.
• This paper will determine regional groupings in North Sumatra 2020 based on significant predictors in modeling morbidity rates.

## Graphical abstract

**Figure:**
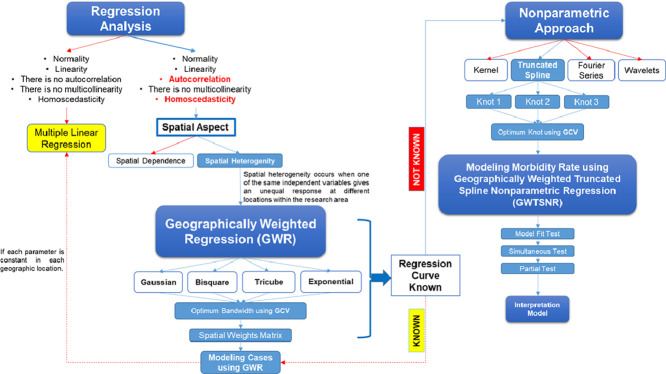

Specifications Table

**Table:** 

Subject area	Mathematics and Statistics
More specific subject area	Statistics: Nonparametric Regression, Spatial Regression.
Name of your method	Geographically Weighted Truncated Spline Nonparametric Regression (GWTSNR)
Name and reference of original method	Original Method
	Geographically Weighted Regression with Spline Approach.
	Reference
	Sifriyani, S.H. Kartiko, I.N. Budiantara, and Gunardi, Geographically Weighted Regression with Spline Approach. Far East Journal of Mathematical Sciences, 101 (6) (2017) 1183-1196. DOI: 10.17654/MS101061183
Resource availability	Morbidity rate data (Y) from The Health Office in North Sumatra and The predictors (X) from the Central Statistics Agency in North Sumatra.

## Method details

### Introduction

Health is a state of complete physical, mental, and social well-being and not merely the absence of disease or infirmity. Health is a state of complete physical, mental, and social well-being and not merely the absence of disease or infirmity. Therefore, the health indicators in the area can be measured by the number of people who experience illness or contract a disease. Illness or health complaints in Indonesia are referred to as morbidity. There are many uses for morbidity rates in a country. Morbidity statistics measure a country's level of health and the provision of health facilities. This data can be used to measure the extent to which medical facilities are utilized and can assist in investigating the pattern of disease occurrence [1].

According to the Performance Report of the Government Agencies of the Health Office of North Sumatra Province in 2020, it is explained that the same as the national condition in Indonesia, in the last five years, the Morbidity Rate in North Sumatra was 11.84% in 2015, decreased to 11.15% in 2016, increased again to 11.17% in 2017 then again reduced to 11.03% in 2018, but in 2019 increased to 11.97% and increased again in 2020 to 12.24% [2]. The morbidity rate has a more critical role than the mortality rate. Because if the morbidity rate is high, it will trigger death, then a high mortality rate, so that life expectancy in an area will be below. In general, it can be said that the realization of optimal public health is one element of the welfare of the national goal, namely the ability to live healthily for every resident. The morbidity rate is data influenced by spatial effects. Spatial data is dependent data where measurement data at other locations affect data at one location [3]. As a result, the spatial data is unsuitable for solving using linear regression analysis because it will produce an inaccurate model. In linear regression analysis, it is assumed that the error variance is fixed (homoscedasticity) and there is no dependence between errors (autocorrelation) at each observation location. Suppose the results of the regression analysis show the existence of heteroscedasticity and autocorrelation. In that case, it can be indicated that the parameters of the regression model are influenced by other factors, namely geographical factors. Therefore, in spatial data analysis, geographical factors are essential in determining the weights to be used [4]. One spatial regression method that can model spatial data is Geographically Weighted Regression (GWR). GWR was first introduced by Fotheringham in 1967 [5]. In GWR, each parameter is calculated at each location point, resulting in a parameter estimator that can only be used to predict each point or location where the data is observed and concluded. Research using the GWR theory was carried out by Brunsdon et al. (1996), Crespo et al. (2007), Leung et al. (2000a), and Leung et al. (2000b) [6], [7], [8], [9]. The development of GWR has also been carried out by statisticians such as Yu (2010), Wrenn and Sam (2014), and Zuhdi (2017) [10], [11], [12]. The development of GWR carried out by statisticians is still in a linear form. But in reality, not all data is known to have a clear relationship pattern, or the regression curve is unknown [13]. So nonparametric regression is an alternative approach to be used in the cases. Several kinds of nonparametric regression models are often discussed as Spline (Budianatara, 2002; Budiantara et al., 1997; Green and Silverman, 1994; Wahba, 1990), Kernel (Hardle, 1990), Fourier series (Antoniadis, 1994) and Wavelets (Antoniadis, 2001) [14], [15], [16], [17], [18], [19], [20]. A spline is a segmented polynomial that has flexibility properties. Spline is very dependent on the knot point. Truncated Spline is a segmented polynomial model that allows adapting effectively to the local characteristics of the data. Thus, Sifriyani et al. (2017) developed a Geographically Weighted Truncated Spline Nonparametric Regression (GWTSNR) model to solve the problem of spatial analysis in which the regression curve is unknown [21].

In this research, the GWTSNR model will be developed by giving more geographical weights using the Fixed Kernel function, namely Fixed Gaussian, Fixed Bisquare, Fixed Tricube, and Fixed Exponential. Furthermore, the Generalized Cross Validation (GCV) method will be used in selecting the best weighting method, which is the development of the Cross Validation (CV) method.

## The main limitations, applicability, and findings

This study will be explained the formation of the GWTSNR model and the resulting parameter estimates. Based on previous research, it has been explained how to build the GWTSNR model, determine parameter estimates, and test hypotheses with its application to the case of the open unemployment rate. In this study, GWTSNR model requires bandwidth and knot points. Previously they only used two kernel functions as geographic weighting, namely Gaussian and Bisquare, and determined the best weighting using Cross Validation (CV). Then they use variations of knots in the model, namely knot 1, knot 2, and knot 3, for each predictor by determining the best knot using Generalized Cross Validation (GCV).

Furthermore, in this research, we will develop the use of the GWTSNR model for the case studies that we will examine by varying the kernel function into four kernel functions, namely Gaussian, Bisquare, Tricube, and Exponential. Furthermore, in determining the best weighting using Generalized Cross Validation (GCV), a development of Cross Validation (CV). Moreover, we will also provide an algorithm for using this model in analyzing the case studies studied.

## Model specifications and estimation procedures

### TSNR model

Nonparametric regression is one of the regression models used to determine the relationship between the response variable and the predictor variable whose regression curve is unknown. It is a very flexible regression model in modeling data patterns [22]. In general, nonparametric regression models can be presented as follows:

(1) $$y_{i}=f\left(x_{i}\right)+\varepsilon_{i},\;i=1,2,3,\ldots ,n$$

$y_{i}$ is a response variable, $x_{i}$ is predictor variables, $f(x_{i})$ is an unknown regression curve or does not follow a particular pattern, and $\varepsilon_{i}∼IID\;N\left(0,\sigma^{2}\right)$. If the regression curve f is an additive model and is approximated by a spline function, the regression model is obtained as follows:

(2) $$y_{i}=\sum_{k=0}^{m}\beta_{k}x_{i}^{k}+\sum_{h=1}^{r}\beta_{m+h}{\left(x_{i}-K_{h}\right)}_{+}^{m}+\varepsilon_{i},i=1,2,3,\ldots ,n$$

where $\beta_{k}$, $\beta_{m+h}$ are real constants with k=0,1,2,…,m, h=1,2,…,r and then the truncated function is as follows

(3) $${\left(x_{i}-K_{h}\right)}_{+}^{m}=\left\{ \begin{array}{c} {\left(x_{i}-K_{h}\right)}^{m},\;x_{i}\geq K_{h}\\ 0,\;\;x_{i}<K_{h} \end{array}\right.$$

Where, $K_{h}$ is a knot point that shows the shape of the behaviour change of the function at certain sub-intervals. And parameter estimation of the TSNR model was carried out using the maximum likelihood method as follows;

(4) $$\hat{\beta }={\left(X^{\prime }X\right)}^{-1}X^{\prime }Y$$

Where

$\hat{\beta }$: Parameters Estimation of TSNR model.
X: Matrics of predictor variables.
Y**:** Vector of the response variable.

### GWR model

Fotheringham first introduced GWR in 1967. It is the development of multiple linear regression. The multiple linear regression model has constant parameters at each observation location, while GWR has local parameters at each observation location. In the GWR model, the relationship between the response variables y and predictor variables $x_{1},x_{2},\ldots ,x_{p}$ at location i as follows:

(5) $$y_{i}=\beta_{0}\left(u_{i},v_{i}\right)+\sum_{k=1}^{p}\beta_{k}\left(u_{i},v_{i}\right)x_{ki}+\varepsilon_{i}$$

And parameter estimation of the GWR model was carried out using the maximum likelihood method as follows;

(6) $$\hat{\beta }={\left(X^{\prime }W_{i}X\right)}^{-1}X^{\prime }W_{i}Y$$

$\hat{\beta }:$ Parameters Estimation of GWR model
X: Matrics of predictor variables
Y: Vector of the response variable.
$W_{i}$**:** Matrics of geographic weights.

## GWTSNR model

GWTSNR is a development of nonparametric regression for spatial data with local parameter estimators for each observation location. Sifriyani, Gunardi, S.H. Kartiko, and I.N. Budiantara developed the method (2017). GWTSNR is a nonparametric regression approach used to solve spatial analysis problems where the regression curve is unknown. The assumptions used in the GWTSNR model have normally distributed errors, zero mean and variance *σ*^2^ at each location (*u_i_, v_i_*). Mathematically the relationship between the response variable *y_i_* and the predictor variable (*x*_1_*_i_, x*_2_*_i_*,…,*x_li_*) at *i*-th location for the model can be expressed as follows (Sifriyani et al., 2017);

(7) $$y_{i}=\beta_{0}\left(u_{i},v_{i}\right)+\sum_{p=1}^{l}\sum_{k=1}^{m}\beta_{pk}\left(u_{i},v_{i}\right)x_{pi}^{k}+\sum_{p=1}^{l}\sum_{h=1}^{r}\delta_{p,m+h}\left(u_{i},v_{i}\right){\left(x_{pi}-K_{ph}\right)}_{+}^{m}+\varepsilon_{i}$$

Eq. (7) is the GWTSNR model of degree m with n areas. The components are described as follows: $y_{i}$ is response variable on the i-th location where i=1,2,…,n, $x_{pi}$ is the p-th predictor variable in the i-th area with p=1,2,…,l, $K_{ph}$ is the h-th knot point on the p-th predictor variable component with h=1,2,…,r, $\beta_{pk}\left(u_{i},v_{i}\right)$ is a parameter of the polynomial component of the GWTSNR, the k-th parameter of the p-th predictor variable in the i-th area, and $\delta_{p,m+h}\left(u_{i},v_{i}\right)$ is a truncated component parameter of GWTSNR, which is the (m+h)-th parameter, at the h knot point and the p-th predictor variable in the i-th area.

## GWTSNR estimation procedures

Next, we will determine the parameter estimates $\tilde{\beta }\left(u_{i},v_{i}\right)$ dan $\tilde{\delta }\left(u_{i},v_{i}\right)$ from the GWTSNR model using Maximum Likelihood Estimation (MLE). The steps are as follows:

a. Determine the probability density function of $y_{i}$.
  (8) $$f\left(y_{i}\right)=\frac{1}{\sqrt{2\pi \sigma^{2}}}\exp\left(-\frac{1}{2\sigma^{2}}{\left[y_{i}-\left(\beta_{0}\left(u_{i},v_{i}\right)+\sum_{p=1}^{l}\sum_{k=1}^{m}\beta_{pk}\left(u_{i},v_{i}\right)x_{pi}^{k}+\sum_{p=1}^{l}\sum_{h=1}^{r}\delta_{p,m+h}\left(u_{i},v_{i}\right){\left(x_{pi}-K_{ph}\right)}_{+}^{m}\right)\right]}^{2}\right)$$

b. Establish a likelihood function.

$$L\left(\tilde{\beta }\left(u_{i},v_{i}\right),\;\tilde{\delta }\left(u_{i},v_{i}\right),\sigma^{2}|\tilde{Y}\right)=\prod_{i=1}^{n}f\left(y_{i}|\tilde{\beta }\left(u_{i},v_{i}\right),\;\tilde{\delta }\left(u_{i},v_{i}\right),\sigma^{2}\right)$$

(9) $$\;={\left(2\pi \right)}^{-\frac{n}{2}}{\left(\sigma^{2}\right)}^{-\frac{n}{2}}\exp\left(-\frac{1}{2\sigma^{2}}{\left(\tilde{Y}-X\tilde{\beta }\left(u_{i},v_{i}\right)-P\tilde{\delta }\left(u_{i},v_{i}\right)\right)}^{{}^{\prime }}\left(\tilde{Y}-X\tilde{\beta }\left(u_{i},v_{i}\right)-P\tilde{\delta }\left(u_{i},v_{i}\right)\right)\right)$$

c. Establish a weighted likelihood function at the j-th location.
  $$f\left(y_{i}\right)=\frac{1}{\sqrt{2\pi \sigma^{2}}}\exp\left(-\frac{1}{2\sigma^{2}}{\left[y_{i}-\left(\beta_{0}\left(u_{i},v_{i}\right)+\sum_{p=1}^{l}\sum_{k=1}^{m}\beta_{pk}\left(u_{i},v_{i}\right)x_{pi}^{k}+\sum_{p=1}^{l}\sum_{h=1}^{r}\delta_{p,m+h}\left(u_{i},v_{i}\right){\left(x_{pi}-K_{ph}\right)}_{+}^{m}\right)\right]}^{2}\right)$$
  (10) $$={\left(2\pi \right)}^{-\frac{n}{2}}{\left(\sigma^{2}\right)}^{-\frac{n}{2}}\exp\left(-\frac{1}{2\sigma^{2}\left(u_{j},v_{j}\right)}\sum_{i=1}^{n}w_{i\left(j\right)}{\left[y_{j}-\left(\beta_{0}\left(u_{j},v_{j}\right)+\sum_{p=1}^{l}\sum_{k=1}^{m}\beta_{pk}\left(u_{j},v_{j}\right)x_{pi}^{k}+\sum_{p=1}^{l}\sum_{h=1}^{r}\delta_{p,m+h}\left(u_{j},v_{j}\right){\left(x_{pi}-K_{ph}\right)}_{+}^{m}\right)\right]}^{2}\right)$$

$w_{i(j)}$: the weighting value at the i-th location to the j-location.

d. Calculating ln from the weighted likelihood function.
  (11) $$\ln L\left(\tilde{\beta }\left(u_{j},v_{j}\right),\;\tilde{\delta }\left(u_{j},v_{j}\right),\sigma^{2}|\tilde{Y}\right)=-\frac{n}{2}\left(\ln\left(2\pi \right)+\sigma^{2}\right)-\frac{1}{2\sigma^{2}}{\left(\tilde{Y}-X\tilde{\beta }\left(u_{j},v_{j}\right)-P\tilde{\delta }\left(u_{j},v_{j}\right)\right)}^{{}^{\prime }}W\left(u_{j},v_{j}\right)\left(\tilde{Y}-X\tilde{\beta }\left(u_{j},v_{j}\right)-P\tilde{\delta }\left(u_{j},v_{j}\right)\right)$$

where $W\left(u_{j},v_{j}\right)=\text{diag}\;\left(w_{1}\left(u_{j},v_{j}\right),w_{2}\left(u_{j},v_{j}\right),\ldots ,w_{n}\left(u_{j},v_{j}\right)\right)$.

e. Maximize ln L

Let

$$\Psi ={\left(\tilde{Y}-X\tilde{\beta }\left(u_{j},v_{j}\right)-P\tilde{\delta }\left(u_{j},v_{j}\right)\right)}^{{}^{\prime }}W\left(u_{j},v_{j}\right)\left(\tilde{Y}-X\tilde{\beta }\left(u_{j},v_{j}\right)-P\tilde{\delta }\left(u_{j},v_{j}\right)\right)$$

$$\begin{array}{ccc} & & \;={\tilde{Y}}^{T}W\left(u_{j},v_{j}\right)\tilde{Y}-{\tilde{Y}}^{\prime }W\left(u_{j},v_{j}\right)X\tilde{\beta }\left(u_{j},v_{j}\right)-\tilde{Y}{}^{\prime }W\left(u_{j},v_{j}\right)P\tilde{\delta }\left(u_{j},v_{j}\right)-\tilde{\beta }{\left(u_{j},v_{j}\right)}^{\prime }X^{\prime }W\left(u_{j},v_{j}\right)\tilde{Y}\\ & & \;\;+\tilde{\beta }{\left(u_{j},v_{j}\right)}^{{}^{\prime }}X^{\prime }W\left(u_{j},v_{j}\right)X\tilde{\beta }\left(u_{j},v_{j}\right)+\tilde{\beta }{\left(u_{j},v_{j}\right)}^{{}^{\prime }}X^{\prime }W\left(u_{j},v_{j}\right)P\tilde{\delta }\left(u_{j},v_{j}\right)-{\left(\tilde{\delta }\left(u_{j},v_{j}\right)\right)}^{\prime }P^{\prime }W\left(u_{j},v_{j}\right)\tilde{Y}\\ & & \;\;+{\left(\tilde{\delta }\left(u_{j},v_{j}\right)\right)}^{{}^{\prime }}P^{\prime }W\left(u_{j},v_{j}\right)X\tilde{\beta }\left(u_{j},v_{j}\right)+{\left(\tilde{\delta }\left(u_{j},v_{j}\right)\right)}^{{}^{\prime }}P^{\prime }W\left(u_{j},v_{j}\right)P\tilde{\delta }\left(u_{j},v_{j}\right) \end{array}$$

(12) $$\begin{array}{ccc} & & \;={\tilde{Y}}^{{}^{\prime }}W\left(u_{j},v_{j}\right)Y-2\tilde{\beta }{\left(u_{j},v_{j}\right)}^{{}^{\prime }}X^{\prime }W\left(u_{j},v_{j}\right)\tilde{Y}-2{\left(\tilde{\delta }\left(u_{j},v_{j}\right)\right)}^{{}^{\prime }}P^{\prime }W\left(u_{j},v_{j}\right)\tilde{Y}\\ & & \;\;+\tilde{\beta }{\left(u_{j},v_{j}\right)}^{{}^{\prime }}X^{\prime }W\left(u_{j},v_{j}\right)X\tilde{\beta }\left(u_{j},v_{j}\right)+2{\left(\tilde{\delta }\left(u_{j},v_{j}\right)\right)}^{{}^{\prime }}P^{\prime }W\left(u_{j},v_{j}\right)X\tilde{\beta }\left(u_{j},v_{j}\right)\\ & & \;\;+{\left(\tilde{\delta }\left(u_{j},v_{j}\right)\right)}^{{}^{\prime }}P^{\prime }W\left(u_{j},v_{j}\right)P\tilde{\delta }\left(u_{j},v_{j}\right) \end{array}$$

Furthermore, to obtain the estimator of $\hat{\tilde{\beta }}\left(u_{j},v_{j}\right)\;$and $\hat{\tilde{\delta }}\left(u_{j},v_{j}\right)$ can be obtained by completing the following optimization:

(13) $$\max_{\tilde{\beta },\tilde{\delta },\sigma^{2}}\left\{\ln L\left(\tilde{\beta }\left(u_{j},v_{j}\right),\;\tilde{\delta }\left(u_{j},v_{j}\right),\sigma^{2}|\tilde{Y}\right)\right\}=\max_{\tilde{\beta },\tilde{\delta },\sigma^{2}}\left\{-\frac{n}{2}\ln\left(2\pi \right)-\frac{n}{2}\ln\left(\sigma^{2}\right)-\frac{1}{2\sigma^{2}}\Psi \right\}$$

In other words, the estimators $\hat{\tilde{\beta }}\left(u_{i},v_{i}\right)\;$and $\hat{\tilde{\delta }}\left(u_{i},v_{i}\right)$ can be obtained with MLE by completing the following optimization:

(14) $$\max_{\tilde{\beta },\tilde{\delta },\sigma^{2}}\left\{\ln L\left(\tilde{\beta }\left(u_{i},v_{i}\right),\;\tilde{\delta }\left(u_{i},v_{i}\right),\sigma^{2}|\tilde{Y}\right)\right\}=\max_{\tilde{\beta },\tilde{\delta },\sigma^{2}}\left\{-\frac{n}{2}\ln\left(2\pi \right)-\frac{n}{2}\ln\left(\sigma^{2}\right)-\frac{1}{2\sigma^{2}}\Psi \right\}$$

Parameters Estimation $\tilde{\beta }\left(u_{i},v_{i}\right)$:

$$\frac{\partial \left(\ln L\left(\tilde{\beta }\left(u_{i},v_{i}\right),\;\tilde{\delta }\left(u_{i},v_{i}\right),\sigma^{2}|\tilde{Y}\right)\right)}{\partial \tilde{\beta }\left(u_{i},v_{i}\right)}=\frac{\partial \left(-\frac{n}{2}\ln\left(2\pi \right)-\frac{n}{2}\ln\left(\sigma^{2}\right)-\frac{1}{2\sigma^{2}}\Psi \right)}{\partial \tilde{\beta }\left(u_{i},v_{i}\right)}=0$$

$$2X^{\prime }W\left(u_{i},v_{i}\right)\tilde{Y}-2X^{\prime }W\left(u_{i},v_{i}\right)X\tilde{\beta }\left(u_{i},v_{i}\right)-2X^{\prime }W\left(u_{i},v_{i}\right)P\tilde{\delta }\left(u_{i},v_{i}\right)=0$$

(15) $$\hat{\tilde{\beta }}\left(u_{i},v_{i}\right)={\left(X^{\prime }W\left(u_{i},v_{i}\right)X\right)}^{-1}\left[X^{\prime }W\left(u_{i},v_{i}\right)\tilde{Y}-X^{\prime }W\left(u_{i},v_{i}\right)P\tilde{\delta }\left(u_{i},v_{i}\right)\right]$$

Parameters Estimation $\tilde{\delta }\left(u_{i},v_{i}\right)$:

$$\frac{\partial \left(\ln L\left(\tilde{\beta }\left(u_{i},v_{i}\right),\;\tilde{\delta }\left(u_{i},v_{i}\right),\sigma^{2}|\tilde{Y}\right)\right)}{\partial \tilde{\delta }\left(u_{i},v_{i}\right)\;}=\frac{\partial \left(-\frac{n}{2}\ln\left(2\pi \right)-\frac{n}{2}\ln\left(\sigma^{2}\right)-\frac{1}{2\sigma^{2}}\Psi \right)}{\partial \tilde{\delta }\left(u_{i},v_{i}\right)\;}=0$$

$$-2P^{\prime }W\left(u_{i},v_{i}\right)\;\tilde{Y}+2P^{\prime }W\left(u_{i},v_{i}\right)\;X\tilde{\beta }\left(u_{i},v_{i}\right)\;+2P^{\prime }W\left(u_{i},v_{i}\right)\;P\tilde{\delta }\left(u_{i},v_{i}\right)\;=0$$

(16) $$\hat{\tilde{\delta }}\left(u_{i},v_{i}\right)\;={\left(P^{T}W\left(u_{i},v_{i}\right)\;P\right)}^{-1}P^{T}W\left(u_{i},v_{i}\right)\;\tilde{Y}-{\left(P^{T}W\left(u_{i},v_{i}\right)\;P\right)}^{-1}P^{T}W\left(u_{i},v_{i}\right)\;X\hat{\tilde{\beta }}\left(u_{i},v_{i}\right)$$

To obtain an estimator $\hat{\tilde{\beta }}\left(u_{i},v_{i}\right)$ that is independent on $\hat{\tilde{\delta }}\left(u_{i},v_{i}\right)$, then substitute Eq. 16 into Eq. 15 as follows.

$$\begin{array}{ccc} \overset{⌢}{\tilde{\beta }}\left(u_{i},v_{i}\right) & = & {\left(X^{\prime }W\left(u_{i},v_{i}\right)X\right)}^{-1}X^{\prime }W\left(u_{i},v_{i}\right)\tilde{Y}-{\left(X^{\prime }W\left(u_{i},v_{i}\right)X\right)}^{-1}X^{\prime }W\left(u_{i},v_{i}\right)P{\left(P^{\prime }W\left(u_{i},v_{i}\right)P\right)}^{-1}P^{\prime }W\left(u_{i},v_{i}\right)\tilde{Y}\\ & & +{\left(X^{\prime }W\left(u_{i},v_{i}\right)X\right)}^{-1}X^{\prime }W\left(u_{i},v_{i}\right)P{\left(P^{\prime }W\left(u_{i},v_{i}\right)P\right)}^{-1}P^{\prime }W\left(u_{i},v_{i}\right)X\overset{⌢}{\tilde{\beta }}\left(u_{i},v_{i}\right) \end{array}$$

$$\begin{array}{ccc} & & \;\hat{\tilde{\beta }}\left(u_{i},v_{i}\right)-{\left(X^{\prime }W\left(u_{i},v_{i}\right)X\right)}^{-1}X^{\prime }W\left(u_{i},v_{i}\right)P{\left(P^{\prime }W\left(u_{j},v_{j}\right)P\right)}^{-1}P^{\prime }W\left(u_{j},v_{j}\right)X\hat{\tilde{\beta }}\left(u_{i},v_{i}\right)\\ & & \;\;={\left(X^{\prime }W\left(u_{i},v_{i}\right)X\right)}^{-1}X^{\prime }W\left(u_{i},v_{i}\right)\tilde{Y}-{\left(X^{\prime }W\left(u_{i},v_{i}\right)X\right)}^{-1}X^{\prime }W\left(u_{i},v_{i}\right)P{\left(P^{\prime }W\left(u_{i},v_{i}\right)P\right)}^{-1}P^{\prime }W\left(u_{i},v_{i}\right)\tilde{Y} \end{array}$$

$$\begin{array}{ccc} & & \;\left[I-{\left(X^{\prime }W\left(u_{i},v_{i}\right)X\right)}^{-1}X^{\prime }W\left(u_{i},v_{i}\right)P{\left(P^{\prime }W\left(u_{j},v_{j}\right)P\right)}^{-1}P{}^{\prime }W\left(u_{j},v_{j}\right)X\right]\hat{\tilde{\beta }}\left(u_{i},v_{i}\right)\\ & & \;\;={\left(X^{\prime }W\left(u_{i},v_{i}\right)X\right)}^{-1}X^{\prime }W\left(u_{i},v_{i}\right)\tilde{Y}-{\left(X^{\prime }W\left(u_{i},v_{i}\right)X\right)}^{-1}X^{\prime }W\left(u_{i},v_{i}\right)P{\left(P{}^{\prime }W\left(u_{i},v_{i}\right)P\right)}^{-1}P{}^{\prime }W\left(u_{i},v_{i}\right)\tilde{Y} \end{array}$$

$$\begin{array}{ccc} \hat{\tilde{\beta }}\left(u_{i},v_{i}\right) & = & {\left[I-{\left(X^{\prime }W\left(u_{i},v_{i}\right)X\right)}^{-1}X^{\prime }W\left(u_{i},v_{i}\right)P{\left(P^{\prime }W\left(u_{j},v_{j}\right)P\right)}^{-1}P^{\prime }W\left(u_{i},v_{i}\right)X\right]}^{-1}\\ & & \left[{\left(X^{\prime }W\left(u_{i},v_{i}\right)X\right)}^{-1}X^{\prime }W\left(u_{i},v_{i}\right)\tilde{Y}-{\left(X^{\prime }W\left(u_{i},v_{i}\right)X\right)}^{-1}X^{\prime }W\left(u_{i},v_{i}\right)P{\left(P^{\prime }W\left(u_{i},v_{i}\right)P\right)}^{-1}P^{\prime }W\left(u_{i},v_{i}\right)\tilde{Y}\right] \end{array}$$

$$\hat{\tilde{\beta }}\left(u_{i},v_{i}\right)=S{\left(X^{\prime }W\left(u_{i},v_{i}\right)X\right)}^{-1}\left[X^{\prime }-X^{\prime }W\left(u_{i},v_{i}\right)P{\left(P^{\prime }W\left(u_{i},v_{i}\right)P\right)}^{-1}P^{\prime }\right]W\left(u_{i},v_{i}\right)\tilde{Y}$$

(17) $$\hat{\tilde{\beta }}\left(u_{i},v_{i}\right)=A\left(K\right)\tilde{Y}$$

Where,

$$A\left(K\right)=S{\left(X^{\prime }W\left(u_{i},v_{i}\right)X\right)}^{-1}\left[X^{\prime }-X^{\prime }W\left(u_{i},v_{i}\right)P{\left(P^{\prime }W\left(u_{i},v_{i}\right)P\right)}^{-1}P^{\prime }\right]W\left(u_{i},v_{i}\right)$$

$$S={\left[I-{\left(X^{\prime }W\left(u_{i},v_{i}\right)X\right)}^{-1}X^{\prime }W\left(u_{i},v_{i}\right)P{\left(P^{\prime }W\left(u_{j},v_{j}\right)P\right)}^{-1}P^{\prime }W\left(u_{i},v_{i}\right)X\right]}^{-1}.$$

To obtain an estimator $\hat{\tilde{\delta }}\left(u_{i},v_{i}\right)$ that is independent on $\hat{\tilde{\beta }}\left(u_{i},v_{i}\right)$, then substitute Eq. 15 into Eq. 16 as follows.

$$\begin{array}{ccc} \hat{\tilde{\delta }}\left(u_{i},v_{i}\right) & = & {\left(P^{\prime }W\left(u_{i},v_{i}\right)P\right)}^{-1}P^{\prime }W\left(u_{i},v_{i}\right)\tilde{Y}-{\left(P^{\prime }W\left(u_{i},v_{i}\right)P\right)}^{-1}P^{\prime }W\left(u_{i},v_{i}\right)X\left({\left(X^{\prime }W\left(u_{i},v_{i}\right)X\right)}^{-1}X^{\prime }W\left(u_{i},v_{i}\right)\tilde{Y}\right)\\ & & +{\left(P^{\prime }W\left(u_{i},v_{i}\right)P\right)}^{-1}P^{\prime }W\left(u_{i},v_{i}\right)X\left({\left(X^{\prime }W\left(u_{i},v_{i}\right)X\right)}^{-1}X^{\prime }W\left(u_{i},v_{i}\right)P\hat{\tilde{\delta }}\left(u_{i},v_{i}\right)\right) \end{array}$$

$$\begin{array}{ccc} & & \;\hat{\tilde{\delta }}\left(u_{i},v_{i}\right)-{\left(P^{\prime }W\left(u_{i},v_{i}\right)P\right)}^{-1}P{}^{\prime }W\left(u_{i},v_{i}\right)X{\left(X^{\prime }W\left(u_{i},v_{i}\right)X\right)}^{-1}X^{\prime }W\left(u_{i},v_{i}\right)P\hat{\tilde{\delta }}\left(u_{i},v_{i}\right)={\left(P^{\prime }W\left(u_{i},v_{i}\right)P\right)}^{-1}P^{\prime }W\left(u_{i},v_{i}\right)\tilde{Y}\\ & & \;\;-{\left(P{}^{\prime }W\left(u_{i},v_{i}\right)P\right)}^{-1}P{}^{\prime }W\left(u_{i},v_{i}\right)X{\left(X{}^{\prime }W\left(u_{i},v_{i}\right)X\right)}^{-1}X{}^{\prime }W\left(u_{i},v_{i}\right)\tilde{Y} \end{array}$$

$$\begin{array}{ccc} \hat{\tilde{\delta }}\left(u_{i},v_{i}\right) & = & {\left[I-{\left(P^{\prime }W\left(u_{i},v_{i}\right)P\right)}^{-1}P^{\prime }W\left(u_{i},v_{i}\right)X{\left(X^{\prime }W\left(u_{i},v_{i}\right)X\right)}^{-1}X^{\prime }W\left(u_{i},v_{i}\right)P\right]}^{-1}{\left(P^{\prime }W\left(u_{i},v_{i}\right)P\right)}^{-1}P^{\prime }W\left(u_{i},v_{i}\right)\tilde{Y}\\ & & -{\left(P^{\prime }W\left(u_{i},v_{i}\right)P\right)}^{-1}P^{\prime }W\left(u_{i},v_{i}\right)X{\left(X^{\prime }W\left(u_{i},v_{i}\right)X\right)}^{-1}X^{\prime }W\left(u_{i},v_{i}\right)\tilde{Y} \end{array}$$

$$\hat{\tilde{\delta }}\left(u_{i},v_{i}\right)=R\left[{\left(P^{\prime }W\left(u_{i},v_{i}\right)P\right)}^{-1}P{}^{\prime }W\left(u_{i},v_{i}\right)\tilde{Y}-{\left(P^{\prime }W\left(u_{i},v_{i}\right)P\right)}^{-1}P^{\prime }W\left(u_{i},v_{i}\right)X{\left(X^{\prime }W\left(u_{i},v_{i}\right)X\right)}^{-1}X^{\prime }W\left(u_{i},v_{i}\right)\tilde{Y}\right]$$

$$\hat{\tilde{\delta }}\left(u_{i},v_{i}\right)=R{\left(P^{\prime }W\left(u_{i},v_{i}\right)P\right)}^{-1}\left[P^{\prime }-P^{\prime }W\left(u_{i},v_{i}\right)X{\left(X^{\prime }W\left(u_{i},v_{i}\right)X\right)}^{-1}X^{\prime }\right]W\left(u_{i},v_{i}\right)\tilde{Y}$$

(18) $$\hat{\tilde{\delta }}\left(u_{i},v_{i}\right)=B\left(K\right)\tilde{Y}$$

Where,

$$B\left(K\right)=R{\left(P^{\prime }W\left(u_{i},v_{i}\right)P\right)}^{-1}\left[P^{\prime }-P^{\prime }W\left(u_{i},v_{i}\right)X{\left(X^{\prime }W\left(u_{i},v_{i}\right)X\right)}^{-1}X^{\prime }\right]W\left(u_{i},v_{i}\right)$$

$$R={\left[I-{\left(P^{\prime }W\left(u_{i},v_{i}\right)P\right)}^{-1}P^{\prime }W\left(u_{i},v_{i}\right)X{\left(X^{\prime }W\left(u_{i},v_{i}\right)X\right)}^{-1}X^{\prime }W\left(u_{i},v_{i}\right)P\right]}^{-1}.$$

## Optimum knot point determination

The knot point is a joint point where there is a change in the behavior pattern of the function or curve. However, the number of knot points will also affect the complexity of the model with the many parameters used so that the proper method is needed to determine the optimal knot point. Optimal knot points can be obtained using the Generalized Cross Validation (GCV). The GCV method is generally defined as follows [22,23].

(19) $$GCV\;\left(K_{1},K_{2},K_{3},\ldots ,K_{r}\right)=\frac{MSE\;\left(K_{1},K_{2},K_{3},\ldots ,K_{r}\right)}{{\left(n^{-1}tr\left[I-A\left(K_{1},K_{2},K_{3},\ldots ,K_{r}\right)\right]\right)}^{2}}$$

Where,

I: the identity matrics
n: the number of observations
$K_{1},K_{2},K_{3},\ldots ,K_{r}$: knot points
$MSE(K_{1},K_{2},K_{3},\ldots ,K_{r})$: Mean Square Error of TSNR Model.

## Optimum bandwidth and geographic weights matrics determination

The role of weights in the GWR model is critical because this weighting value represents the location of the observation data with others. Lesage (2001) introduced several weighting methods using Kernel functions, including the Gaussian Kernel, the Exponential Kernel, the Bisquare Kernel, and the Tricube Kernel [24].

i. *Gaussian*
  (20) $$w_{j}\left(u_{i},v_{i}\right)=\phi \left(\frac{d_{ij}}{\sigma h}\right),\;j=1,2,3,\ldots ,n$$

Where ϕis the standard normal density function and denotes the standard deviation of the distance vector $d_{ij}$.

ii. Exponential
  (21) $$w_{j}\left(u_{i},v_{i}\right)=\sqrt{exp\left(-{\left(\frac{d_{ij}}{h}\right)}^{2}\right)}$$

$d_{ij}$: the distance from i-th location to j-th location, and h is the bandwidth value, which is a function smoothing parameter value whose value is always positive.

iii. *Bisquare*
  (22) $$w_{j}\left(u_{i},v_{i}\right)=\{\begin{array}{c} {\left(1-{\left(\frac{d_{ij}}{h}\right)}^{2}\right)}^{2},\;d_{ij}\leq h\\ 0,\;d_{ij}>h \end{array}$$
iv. *Tricube*
  (23) $$w_{j}\left(u_{i},v_{i}\right)=\{\begin{array}{c} {\left(1-{\left(\frac{d_{ij}}{h}\right)}^{3}\right)}^{3},\;d_{ij}\leq h\\ 0,\;d_{ij}>h \end{array}$$

*where* $d_{ij}=\sqrt{{\left(u_{i}-u_{i}\right)}^{2}+{\left(v_{i}-v_{j}\right)}^{2}}$: Euclidean Distance between location $\left(u_{i},v_{i}\right)$ to location $\left(u_{j},v_{j}\right)$ And is a known non-negative parameter called bandwidth or smoothing parameter. The optimum bandwidth can be determined using GCV, which is as follows.

(24) $$GCV=\frac{n^{-1}\sum_{i=1}^{n}{\left[y_{i}-{\hat{y}}_{i}\right]}^{2}}{{\left\{1-\frac{tr\left(H\left(h\right)\right)}{n}\;\right\}}^{2}}$$

Where

GCV: GCV value on bandwidth
tr(H(h)): the sum of the main diagonal elements of the n×n weight matrix
In this study, in selecting the optimum bandwidth using Generalized Cross Validation (GCV). The optimum bandwidth is chosen by finding the smallest GCV. The smallest GCV is generated from the model that has the slightest error.

## Spatial heterogeneity test

Differences in characteristics between observation points cause spatial heterogeneity. Identification of spatial homogeneity can be made by using the *Breusch-Pagan* test. Hypotheses used in the *Breusch-Pagan* test [25]:

$H_{0}$: $\sigma_{1}^{2}=\sigma_{2}^{2}=\ldots =\sigma_{n}^{2}=\sigma^{2}$ (homoscedasticity)
$H_{1}$: At least there is one $\sigma_{i}^{2}\neq \sigma^{2}$ (heteroscedasticity)

## Test statistics

(25) $$BP=\frac{1}{2}f^{T}Z{\left(Z^{T}Z\right)}^{-1}Z^{T}f$$

where

$f={\left(f_{1},f_{2},\ldots ,f_{n}\right)}^{T}$; $f_{i}=\left(\frac{\varepsilon_{i}^{2}}{\sigma^{2}}-1\right)$, $\varepsilon_{i}=y_{i}-{\hat{y}}_{i}$; Z is a matrix containing vectors that have been standardized for each observation. Reject $H_{0}$ if $BP>\chi_{\left(p\right)}^{2}$or p−value<α where p is the number of predictors.

## Model fit significance test

The model fit significance test determines whether the GWTSNR model is better than the global model. The following hypothesis is used [26].

$H_{0}$: $\beta_{pk}\left(u_{i},v_{i}\right)=\beta_{pk}$ and $\delta_{p,m+h}\left(u_{i},v_{i}\right)=\delta_{p,m+h}$
p=1,2,…,l;k=1,2,…,m;h=1,2,…,r;i=1,2,…,n
$H_{1}$: At least one $\beta_{pk}\left(u_{i},v_{i}\right)\neq \beta_{pk}$ or $\delta_{p,m+h}\left(u_{i},v_{i}\right)\neq \delta_{p,m+h}$
p=1,2,…,l;k=1,2,…,m;h=1,2,…,r;i=1,2,…,n

The test statistics used

$$S=I-Q{\left(Q^{\prime }Q\right)}^{-1}Q^{\prime }$$

Where,

$$S=I-Q{\left(Q^{\prime }Q\right)}^{-1}Q^{\prime }$$

n−lm−1: The degrees of freedom ${\tilde{Y}}^{\prime }S\tilde{Y}$

$$D\left(u_{i},v_{i}\right)=\left(I-W\left(u_{i},v_{i}\right)Q{\left(Q^{\prime }W\left(u_{i},v_{i}\right)Q\right)}^{-1}Q^{\prime }\right)\left(I-Q{\left(Q^{\prime }W\left(u_{i},v_{i}\right)Q\right)}^{-1}Q^{\prime }W\left(u_{i},v_{i}\right)\right)$$

(26) $$tr\left({\left(I-\xi \right)}^{\prime }\left(I-\xi \right)\right):\text{The}\;\text{degrees}\;\text{of}\;\text{freedom}\;{\tilde{Y}}^{\prime }D\left(u_{i},v_{i}\right)\tilde{Y}$$

Rejection Criteria, $H_{0}$ is rejected if

(27) $${}^{V>F}\left(\left(n-lm-1\right),\frac{{\left(tr\left({\left(I-\xi \right)}^{\prime }\left(I-\xi \right)\right)\right)}^{2}}{\sum_{i=1}^{n}\lambda_{i}^{2}}\right)$$

## Simultaneous parameter significance test

A simultaneous test was conducted to determine the significance of the regression model parameters together. The form of the accompanying test hypothesis is as follows [27].

$H_{0}:\beta_{11}\left(u_{i},v_{i}\right)=\beta_{12}\left(u_{i},v_{i}\right)=\ldots =\beta_{lm}\left(u_{i},v_{i}\right)=\delta_{1,m+1}\left(u_{i},v_{i}\right)=\delta_{1,m+2}\left(u_{i},v_{i}\right)=\ldots =\delta_{1,m+r}\left(u_{i},v_{i}\right)=0,i=1,2,\ldots ,n$
$H_{1}$: At least there is one $\beta_{pk}\left(u_{i},v_{i}\right)\neq 0$ or $\delta_{p,m+h}\left(u_{i},v_{i}\right)\neq 0$
  p=1,2,…,l;k=1,2,…,m;h=1,2,…,r;i=1,2,…,n

The test statistics used:

(28) $$V^{*}=\frac{{\tilde{Y}}^{\prime }M\left(u_{i},v_{i}\right)\tilde{Y}\;/\;tr\left({\left(I-B_{\omega }\right)}^{\prime }\left(I-B_{\omega }\right)\right)}{{\tilde{Y}}^{\prime }D\left(u_{i},v_{i}\right)\tilde{Y}\;/\;tr\left({\left(I-\xi \right)}^{\prime }\left(I-\xi \right)\right)}∼F_{\left(\frac{{\left(tr\left({\left(I-B_{\omega }\right)}^{\prime }\left(I-B_{\omega }\right)\right)\right)}^{2}}{\sum_{i=1}^{n}\gamma_{i}^{2}},\frac{{\left(tr\left({\left(I-\xi \right)}^{\prime }\left(I-\xi \right)\right)\right)}^{2}}{\sum_{i=1}^{n}\lambda_{i}^{2}}\right)}^{*}$$

Where,

$$M\left(u_{i},v_{i}\right)={\left(I-B_{\omega }\right)}^{{}^{\prime }}\left(I-B_{\omega }\right)$$

$tr({\left(I-B_{\omega }\right)}^{{}^{\prime }}\left(I-B_{\omega }\right))$: The degrees of freedom ${\tilde{Y}}^{\prime }M\left(u_{i},v_{i}\right)\tilde{Y}$
  $$D\left(u_{i},v_{i}\right)=\left(I-W\left(u_{i},v_{i}\right)Q{\left(Q^{\prime }W\left(u_{i},v_{i}\right)Q\right)}^{-1}Q^{\prime }\right)\left(I-Q{\left(Q^{\prime }W\left(u_{i},v_{i}\right)Q\right)}^{-1}Q^{\prime }W\left(u_{i},v_{i}\right)\right)$$
$tr({\left(I-\xi \right)}^{{}^{\prime }}\left(I-\xi \right))$: The degrees of freedom ${\tilde{Y}}^{\prime }D\left(u_{i},v_{i}\right)\tilde{Y}$

Rejection Criteria, $H_{0}$ is rejected if

(29) $$V^{*}>F_{\left(\frac{{\left(tr\left({\left(I-B_{\omega }\right)}^{{}^{\prime }}\left(I-B_{\omega }\right)\right)\right)}^{2}}{\sum_{i=1}^{n}\gamma_{i}^{2}},\frac{{\left(tr\left({\left(I-\xi \right)}^{{}^{\prime }}\left(I-\xi \right)\right)\right)}^{2}}{\sum_{i=1}^{n}\lambda_{i}^{2}}\right)}^{*}$$

## Partial parameter significance test

Individual testing is carried out to determine whether the individual parameters have a significant effect on the response variable, with the following hypothesis:

$H_{0}:\beta_{pk}\left(u_{i},v_{i}\right)=0$ and $\delta_{p,m+h}\left(u_{i},v_{i}\right)=0$ with p=1,2,…,7;k=1;h=1,2,3;i=1,2,…,n
$H_{1}$: At least there is one $\beta_{pk}\left(u_{i},v_{i}\right)\neq 0$ or $\delta_{p,m+h}\left(u_{i},v_{i}\right)\neq 0,$ p=1,2,…,7;k=1;h=1,2,3;i=1,2,…,n

The test statistics used:

(30) $$t=\frac{\hat{\tilde{\eta }}\left(u_{i},v_{i}\right)}{SE\left(\hat{\tilde{\eta }}\left(u_{i},v_{i}\right)\right)}=\frac{\hat{\tilde{\eta }}\left(u_{i},v_{i}\right)}{\sqrt{g_{kk}}}$$

Where,$\;\sqrt{g_{kk}}\;$is the k+1 diagonal element of the matrix ${\left(Q^{\prime }W\left(u_{i},v_{i}\right)Q\right)}^{-1}{\hat{\sigma }}^{2}\left(u_{i},v_{i}\right)$. The test statistic for the GWTSNR model in Eq. 30 will follow a t distribution with degrees of freedom n−1 and a significance level of α. The rejection area will reject $H_{0}$ if the value $|t|>t_{\left(\frac{\alpha }{2},\left(n-1\right)\right)}$ or p−value<α, which means that the parameter has a significant effect on the model (Tables 2 and 5).

## The research steps

The steps of analysis in the research are as follows:

a. Describe the morbidity rate in North Sumatra and its predictors.
b. Make a scatterplot between the morbidity rate and each predictor to determine the relationship pattern.
c. Do spatial heterogeneity tests using the Breusch-Pagan method.
d. Calculates the Euclidean distance between the i-th location $(u_{i},v_{i})\;$and the j-th location $\left(u_{j},v_{j}\right)$
e. Determine the best weighting of the kernel functions, namely Gaussian, Bisquare, Tricube, and Exponential, based on the minimum GCV value.
f. Choose the optimum knot point based on the minimum GCV value.
g. Get the best GWTSNR model.
h. Test the fit model hypothesis between the GWTSNR model and the TSNR model.
i. Determine parameter significance tests simultaneously and partially.
j. Interpret the GWTSNR model.
k. Map 33 districts/cities in North Sumatra based on significant predictor variables.

## Data

### Morbidity rate

Morbidity is a condition where a person is said to be sick if the perceived health complaints cause disruption of daily activities, namely, unable to carry out work activities, take care of the household, and carry out normal activities as usual. The formula for calculating the morbidity rate is as follows [28],

(31) $$\text{AM}=\frac{\text{JKPP}}{\text{JP}}\times \;100$$

where,

AM: Morbidity Rate

JKPP: The number of people who experience health complaints and disruption of activities

JP: Total population

The morbidity rate in an area is affected by some factors. The determinant factors of morbidity are social, economic, and cultural factors [29]. Based on Wulandari (2017), it was found that population density, the average length of schooling, poverty percentage, regional minimum wage, percentage of open defecation households, and percentage of households with a distance from drinking water sources to sewage storage > 10 meters significantly affected to the morbidity rate in East Java [30]. Based on Hanum (2013), using the Multivariate Geographically Weighted Regression model, it was found that life expectancy, illiteracy rate, percentage of the population with protected water sources from wells, percentage of the population seeking outpatient treatment at health workers, percentage of the population with distance sources of drinking water to sewage storage > 10 meters and the percentage of the population with a monthly per capita expenditure of 200,000 to 299,999 for nutritious food significantly affected on morbidity rates [31]. According to Gordon (1954), the morbidity rate was influenced by environmental factors consisting of the biological environment, the physical environment, the socio-economic environment, maternal education level, and health services [32].

Based on the description above, in this research, several predictor variables were used, which were thought to have an effect on the morbidity rate in North Sumatra in 2020. The variables are followed as follows:

Y: Morbidity Rate

$X_{1}$: Poverty Percentage

$X_{2}$: Percentage of Households with Access to Proper Sanitation

$X_{3}$: Population Density

$X_{4}$: Open Unemployment Rate

$X_{5}$: General Hospitals

$X_{6}$: Percentage of Households with Access to Resources Adequate Drinking Water

$X_{7}$: Average Length of School

The data used in this research is secondary data. Morbidity rate data is accessed from the official website of the North Sumatra Provincial Health Office in a publication with the title Government Agency Performance Report of the North Sumatra Provincial Health Office 2020. And all predictors that are considered to affect morbidity rates are accessed from the website of the Central Bureau of Statistics (BPS) North Sumatra or contained in the BPS North Sumatra publication with the title North Sumatra Province in Figures 2021. The research units used are 33 districts/cities in North Sumatra province.

## Characteristics of morbidity rates in North Sumatra

**Fig. 1:**
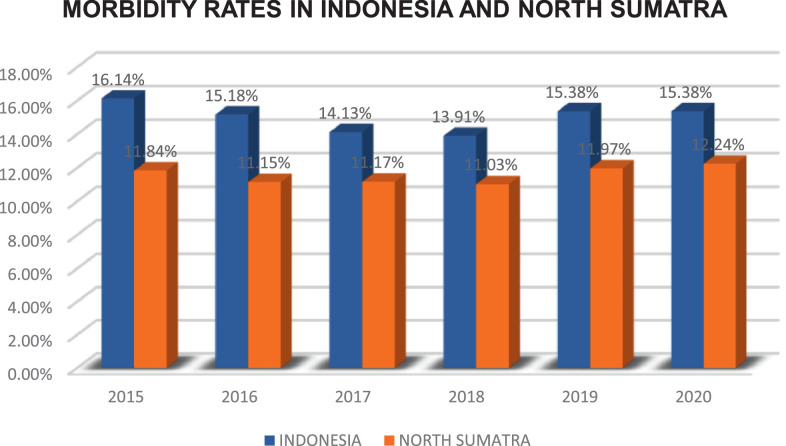
Morbidity Rates in Indonesia and North Sumatra 2015 – 2020. It can be seen that for a period of six years, from 2015 to 2020, the morbidity rates in North Sumatra Province were consistently below the national figure. All variables ranging from the response variable to the seven predictor variables that are thought to affect the average, variance to the minimum, and maximum values are calculated.

**Fig. 2:**
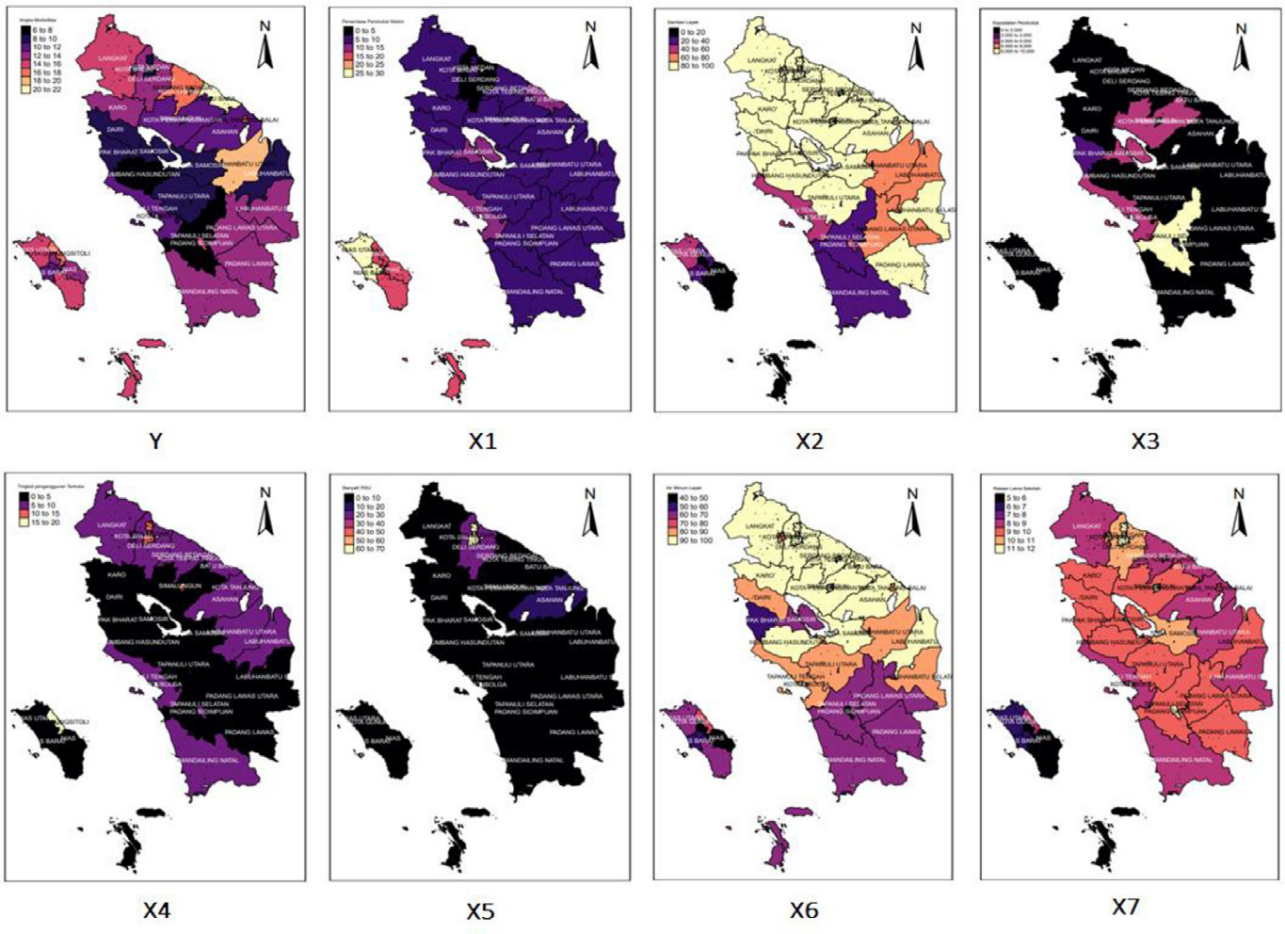
Description information based on variable data mapping. The lowest Y is in Humbang Hasundutan Regency, and the highest Y is in Batubara Regency. The lowest $X_{1}\;$is in Deli Serdang Regency, and the highest $X_{1}$ is in West Nias Regency. The lowest $X_{2}$ is in South Nias Regency, and the highest $X_{2}$ is in Binjai City. The lowest $X_{3}$ is in Pakpak Bharat Regency, and the highest $X_{3}$ is in Medan City. The lowest $X_{4}$ is in Humbang Hasundutan Regency, and the highest $X_{4}$ is in Gunungsitoli City. The lowest $X_{5}\;$is in West Nias Regency, and the highest $X_{5}$ is in Medan City. The lowest $X_{6}$ is in Padang Sidimpuan Regency, and the highest $X_{6}$ is Pematangsiantar City. And the lowest $X_{7}$ is Nias, and the highest $X_{7}$ is in Medan City.

**Fig. 3:**
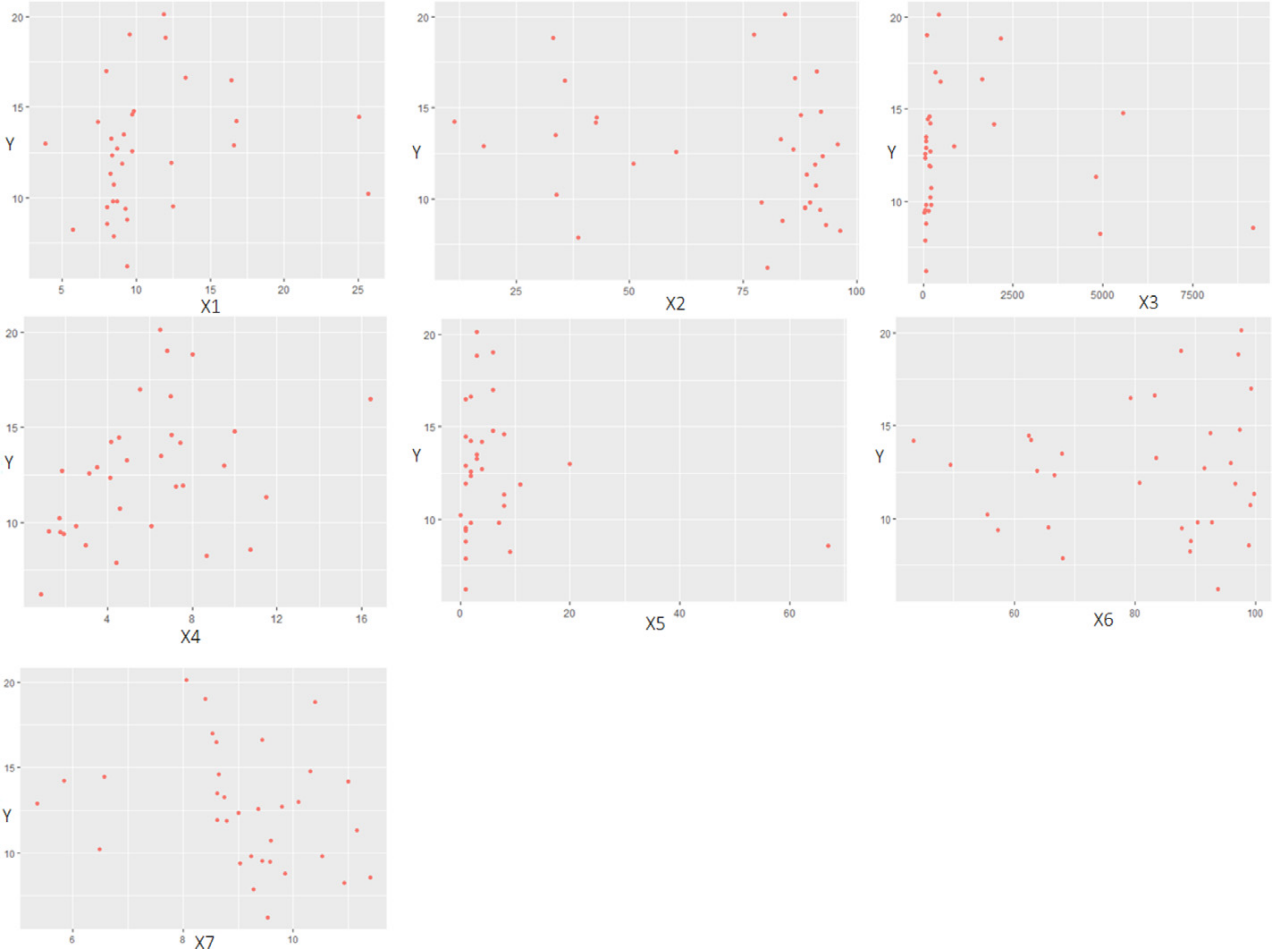
Scatterplot between Morbidity Rate and Predictors. The plot between the variable morbidity rate with all predictors does not form or follow a certain pattern. So that all predictors are included in nonparametric components.

North Sumatra is the second-largest province on Sumatra Island. The population of North Sumatra in 2020 reached 14,799,361 people. 14,799,361 people inhabited the North Sumatra area of 72,981.23 km², and the average population density of North Sumatra was 202.78 people per square kilometer. In 2020 the morbidity rate in North Sumatra reached 12.24. It means that there are 12 out of 100 residents in North Sumatra who experience illness complaints (Fig. 1, Fig. 2, Fig. 3).

The Performance Report of the Government Agencies of the Health Office of North Sumatra Province in 2020 is explained similarly to national conditions. In the last five years, the Sickness Rate in North Sumatra was 11.84% in 2015, decreasing to 11.15% in 2015. 2016, to 11.17% in 2017, then decreased to 11.03% in 2018, but in 2019 it increased to 11.97% and increased again in 2020 to 12.24%, as shown in the following graph

The results of the calculation of descriptive statistics can be presented in Table 1 below.

**Table 1:** Factors that affect morbidity rates in North Sumatra 2020.

Variables	Mean	Minimum	Maximum	Variance
Y	12.56	6.22	20.13	11.74972973
$X_{1}$	10.8	3.88	25.69	22.16703409
$X_{2}$	70.87	11.48	96.2	685.6326
$X_{3}$	1065.80	42.97	73.55	4329029.026
$X_{4}$	5.77	0.84	16.41	11.8475
$X_{5}$	5.94	0	67	136.6837
$X_{6}$	81.37	42.39	99.71	275.9497
$X_{7}$	9.1	5.36	11.39	2.0435

Tabel 1 provides the descriptive statistics of Variables used in the research.

**Table 2:** VIF Value of Independent Variable

Variables	VIF
$X_{1}$	2.852
$X_{2}$	2.617
$X_{3}$	3.814
$X_{4}$	1.774
$X_{5}$	2.478
$X_{6}$	2.081
$X_{7}$	3.698

It can be seen that the VIF value < 10 in all independent variables, and it can be concluded that there is no multicollinearity between the predictor variables used in this study so that the predictor variables in this study can be used in the formation of a regression model.

**Table 3:** Spatial Heterogeneity Test

Breusch Pagan	Df	*p-value*	Decision
**34.647**	14	0.00166	Reject H_0_

Table 3 shows that the $BP>\chi_{\left(k\right)}^{2}=\chi_{\left(7\right)}^{2}=14.0671404$ or p−value=0.00166<0.05 then $H_{0}$ is rejected. In other words, the variance between locations is different (heterogeneous), or there are differences in characteristics between one observation point and another.

**Table 4:** Bandwidth Value and GCV

Kernel Function	Bandwidth Valune (*h*)	GCV
Gaussian	3.296679331	156.2569201
Bisquare	1.499684794	109.1468086
Tricube	1.499965502	110.1381101
Exponential	3.296690063	156.2569174

Table 4 shows that there is spatial heterogeneity, with the optimum bandwidth value of 1.499684794 using the **Bisquare Kernel** weighting function based on the minimum GCV value.

**Tabel 5:** Best Knot Point

Knot Point	GCV
**1**	8.227800831
**2**	11.12530693
**3**	10.02368543

Based on the minimum GCV value, the best model is the GWTSNR model with the one-knot point with a GCV value of 8.227800831.

**Table 6:** Results of ANOVA Model Fit

Variation	Sum of Squares	df	Mean Squares	$F_{\mathrm{counted}}\left(V\right)$	*p-value*
**Regression**	94.2863	25	3.7714	9.868	1.7653e-08
**Error**	11.0831	29	0.3822		
**Total**	105.3694	54			

Table 6 shows that using the significance level α=0.05, $F_{tabel(0.05;25,29)}=1.891$ is obtained. Because $F_{counted}\left(V\right)=9.868>F_{tabel}=1.891\;$then $H_{0}$ is rejected.

**Tabel 7:** Results of ANOVA Simultaneous Parameter Significance Test

Variation	Sum of Squares	df	Mean Squares	$F_{\mathrm{hitung}}\left(V\right)$	*p-value*
**Regression**	302.3977	29	10.4275	27.2845	2.2427e-14
**Error**	11.0831	29	0.3822		
**Total**	313.4808	58			

Table 7 shows that using the significance level α=0.05, $F_{tabel(0.05;29,29)}=1.861$ is obtained. Because $F_{hitung}\left(V^{*}\right)=27.2845>F_{tabel}=1.861$ then $H_{0}$ is rejected.

Furthermore, a mapping of the data information used will be given as follows.

## Results and analysis

### Data patterns between morbidity rates and predictors

Next, a scatterplot of the morbidity rate and the factors that influence it will be presented to see the pattern of relationships between the dependent variables on all independent variables. If the resulting plot forms a certain pattern, then parametric regression is good. Nonparametric regression is appropriate if it does not follow a certain pattern.

## Multicollinearity test

Ragnar Frisch first coined the term multicollinearity. The multicollinearity test is a requirement for all causality (regression) hypothesis tests. Multicollinearity will be detected if the value of $VIF_{k}\left(u_{i},v_{i}\right)>10$. The value of$\;VIF_{k}\left(u_{i},v_{i}\right)$ is stated as follows:

(32) $$VIF_{k}\left(u_{i},v_{i}\right)=\frac{1}{1-R_{k}^{2}\left(u_{i},v_{i}\right)}$$

where $R_{k}^{2}\left(u_{i},v_{i}\right)$ is the coefficient of determination of the k-th variable at the i-th location. The following is the VIF value of the seven independent variables used in this study:

## Spatial heterogeneity test and the best weights matrix

The existence of differences in characteristics between location points causes spatial heterogeneity, so spatial weighting is needed. The best spatial weighting is obtained from the bandwidth value, which has the minimum Generalized Cross Validation (GCV) value. The following are the results of spatial heterogeneity testing and the selection of the best bandwidth.

## Best knot point selection

The next step in determining the best model is determining the knot point. The knot point is the point where the data pattern changes. The following table shows the GCV value at each knot point.

## Parameter estimation of morbidity rate model in North Sumatra in 2020

Based on the results of selecting the optimum knot point, the following parameter estimators from the GWTSNR model with the one-knot point.

(33) $$\begin{array}{ccc} {\hat{y}}_{i} & = & {\hat{\beta }}_{0}\left(u_{i},v_{i}\right)+{\hat{\beta }}_{11}\left(u_{i},v_{i}\right)x_{1i}+{\hat{\beta }}_{21}\left(u_{i},v_{i}\right)x_{2i}+{\hat{\beta }}_{31}\left(u_{i},v_{i}\right)x_{3i}+{\hat{\beta }}_{41}\left(u_{i},v_{i}\right)x_{4i}+{\hat{\beta }}_{51}\left(u_{i},v_{i}\right)x_{5i}\\ & & +{\hat{\beta }}_{61}\left(u_{i},v_{i}\right)x_{6i}+{\hat{\beta }}_{71}\left(u_{i},v_{i}\right)x_{7i}+{\hat{\delta }}_{11}\left(u_{i},v_{i}\right){\left(x_{1i}-K_{11}\right)}_{+}+{\hat{\delta }}_{21}\left(u_{i},v_{i}\right){\left(x_{2i}-K_{21}\right)}_{+}\\ & & +{\hat{\delta }}_{31}\left(u_{i},v_{i}\right){\left(x_{3i}-K_{31}\right)}_{+}+{\hat{\delta }}_{41}\left(u_{i},v_{i}\right){\left(x_{4i}-K_{41}\right)}_{+}+{\hat{\delta }}_{51}\left(u_{i},v_{i}\right){\left(x_{5i}-K_{51}\right)}_{+}+{\hat{\delta }}_{61}\left(u_{i},v_{i}\right){\left(x_{6i}-K_{61}\right)}_{+}\\ & & +\;\;{\hat{\delta }}_{71}\left(u_{i},v_{i}\right){\left(x_{7i}-K_{71}\right)}_{+} \end{array}$$

The following is the GWTSNR model, which is written as an example of the 30th location, namely Medan City.

(34) $$\begin{array}{ccc} {\hat{y}}_{30} & = & -0.091-19.077x_{1;30}+0.450x_{2;30}-0.007x_{3;30}+26.111x_{4;30}+8.749x_{5;30}+2.092x_{6;30}\\ & & -1.853x_{7;30}+19.232{\left(x_{1;30}-4.3162\right)}_{+}-0.752{\left(x_{2;30}-13.1744\right)}_{+}+0.009{\left(x_{3;30}-225.9032\right)}_{+}\\ & & -26.463{\left(x_{4;30}-1.1514\right)}_{+}-8.868{\left(x_{5;30}-1.34\right)}_{+}-1.964{\left(x_{6;30}-44.4184\right)}_{+}-2.354{\left(x_{7;30}-5.4806\right)}_{+} \end{array}$$

## Model fit significance test

Test the model suitability hypothesis between GWTSNR model with the TSNR model. The following is the ANOVA table of the model suitability test.

Thus, it can be concluded that there is a significant difference between GWTSNR model and TSNR model.

## Simultaneous parameter significance test

Simultaneous testing is carried out to test the estimation of model parameters simultaneously. The following are the results of the ANOVA simultaneous parameter test.

Thus it can be concluded that there is at least one parameter in the GWTSNR model that is significant to the response variable or, in other words, poverty percentage, the percentage of households who have access to proper sanitation, population density, open unemployment rate, and general hospital, percentage of households with access to resources adequate drinking water, and the average length of school have a simultaneous effect on the morbidity rate in North Sumatra 2020.

## Partial parameter significance test

The calculation results from the partial parameter significance test show that the predictor variables that have an effect differ for each area. This resulted in 8 districts/cities mapping groups based on influential predictor variables. The grouping of districts/cities based on variables significant to the 2020 morbidity rate in North Sumatra is given as follows.

1. The morbidity rates in Nias, Mandailing Natal, Tapanuli Utara, Labuhanbatu, Simalungun, Dairi, Deli Serdang, Langkat, Nias Selatan, Humbang Hasundutan, Samosir, Batu Bara, Padang Lawas Utara, Padang Lawas, Labuhanbatu Selatan, Nias Utara, Nias Barat, Medan City, Padang Sidimpuan, and Gunungsitoli City are affected by $x_{1},x_{2},x_{3},x_{4},x_{5},x_{6},$ and $x_{7}$.
2. The morbidity rates in Karo, Pematangsiantar City, Tebing Tinggi City, and Binjai City are affected by $x_{1},x_{2},x_{4},x_{5},x_{6},$ and $x_{7}$.
3. The morbidity rates in Tapanuli Selatan, Labuhanbatu Utara, and Tanjung Balai City are affected by $x_{1},x_{2},x_{3},x_{5},x_{6},$ and $x_{7}$.
4. The morbidity rate in Serdang Bedagai is affected by $x_{1},x_{2},x_{6},$ and $x_{7}$.
5. The morbidity rates in Toba Samosir and Pakpak Bharat are affected by $x_{1},x_{4}$ and $x_{5}$.
6. The morbidity rate in Sibolga City is affected by $x_{4}$ and $x_{5}$.
7. The morbidity rate in Asahan is affected by $x_{1}$ and $x_{5}$.
8. The morbidity rate in Tapanuli Tengah is affected by $x_{5}$.

**Fig. 4:**
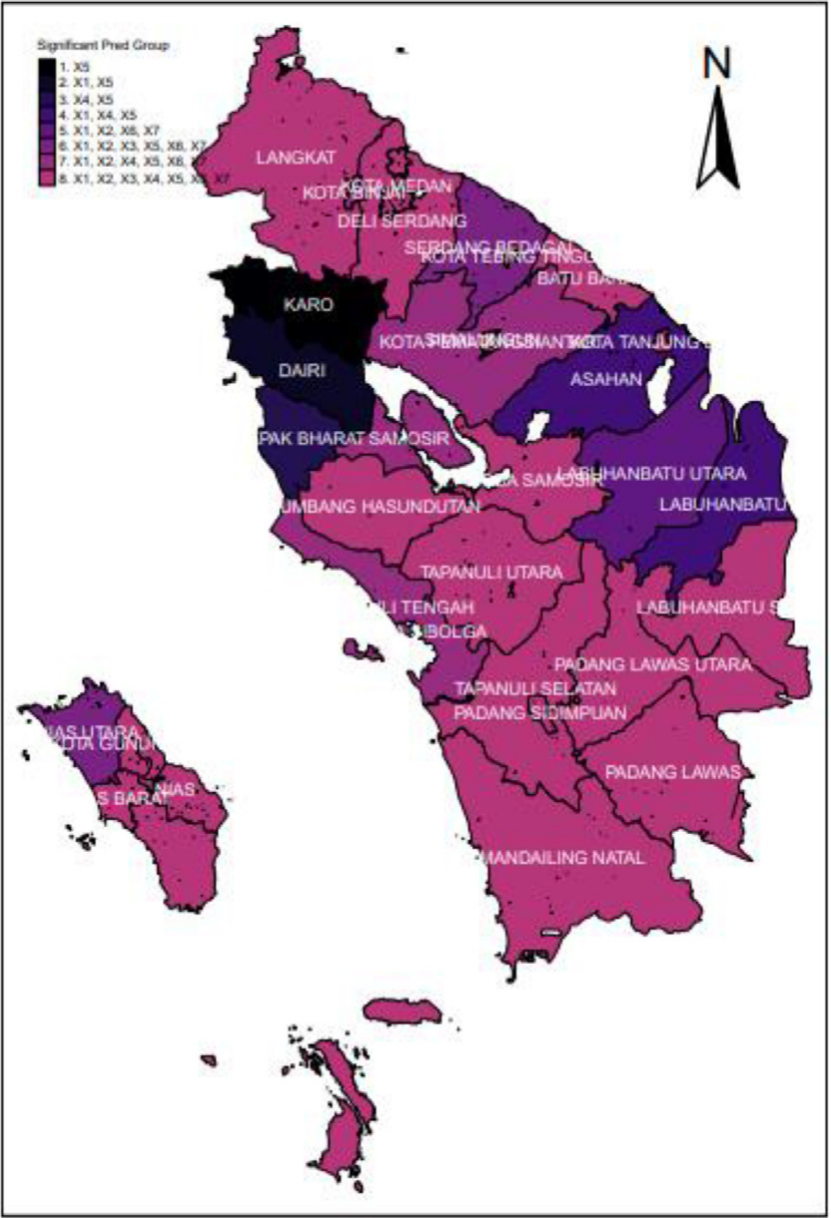
Mapping Morbidity Rates in North Sumatra 2020 based on Significant Variables.

The mapping of Morbidity Rates can be presented in Fig. 4.

## Model interpretation

After doing a partial significance test with 13 regional groups for significant parameters, the best model interpretation is carried out, namely the GWTSNR model with the one-knot point at the 30th location of Medan City in Eq. 34.

The interpretation of the above model is explained as follows.

a. Assuming the predictors ($x_{2}$, $x_{3}$, $x_{4}$, $x_{5}$, $x_{6}$, $x_{7}$) are constant, then the effect of the poverty percentage variable$\;(x_{1})$ on the morbidity rate in 2020 (y) in Medan City can be written as follows.
  $${\hat{y}}_{30}=-19.077x_{1;30}+19.232{\left(x_{1;30}-4.3162\right)}_{+}$$
  (35) $$\;=\left\{ \begin{array}{cc} -19.077x_{1;30}\; & x_{1;30}<4.3162\\ 0.155x_{1;30}+83.009 & x_{1;30}\geq 4.3162 \end{array}\right.$$

Based on the model obtained, it can be interpreted that if the poverty percentage is less than 4.3162%, then every 1% increase in the poverty percentage will reduce the morbidity rate by 19.077%. Meanwhile, if the poverty percentage is more than or equal to 4.3162 %, then every 1% increase in the poverty percentage will increase the morbidity rate by 0.155 %. Because the poverty percentage in Medan City in 2020 is 8.01, if the poverty percentage increases, the morbidity rate will also increase.

b. Assuming the predictors ($x_{1}$, $x_{3}$, $x_{4}$, $x_{5}$, $x_{6}$, $x_{7}$) are constant, then the effect of the percentage of households that have access to proper sanitation $\left(x_{2}\right)$ on the morbidity rate in 2020 (y) in Medan City can be written as follows.
  $${\hat{y}}_{30}=0.450x_{2;30}-0.752{\left(x_{2;30}-13.1744\right)}_{+}$$
  (36) $$\;=\left\{ \begin{array}{cc} 0.450x_{2;30} & ;x_{2;30}<13.1744\\ -0.302x_{2;30}+9.914 & ;x_{2;30}\geq 13.1744 \end{array}\right.$$

Based on the model obtained, it can be interpreted that if the percentage of households with access to proper sanitation is less than 13.1744%, it increases by 1%. The morbidity rate will increase by 0.450%. Meanwhile, if the percentage of households with access to proper sanitation is more than or equal to 13.1744%, it increases by 1%. The morbidity rate will decrease by 0.302%. Because the percentage of households that have access to proper sanitation in Medan City in 2020 is 93.16, if the percentage of households that have access to proper sanitation increases, the morbidity rate will decrease.

c. Assuming the predictors ($x_{1}$, $x_{2}$, $x_{4}$, $x_{5}$, $x_{6}$, $x_{7}$) are constant, then the effect of the population density $\left(x_{3}\right)$ on morbidity rates in 2020 (y) in Medan City can be written as follows.
  $${\hat{y}}_{30}=-0.007x_{3;30}+0.009{\left(x_{3;30}-225.9032\right)}_{+}$$
  (37) $$\;=\left\{ \begin{array}{cc} -0.007x_{3;30} & ;x_{3;30}<225.9032\\ 0.0016x_{3;30}-2.064 & ;x_{3;30}\geq 225.9032 \end{array}\right.$$

Based on the model obtained, it can be interpreted that if the population density is less than 225,903 then it increases by 1 unit, and the morbidity rate will decrease by 0.0075%. Meanwhile, if the population density is more than or equal to 225.903 there is an increase of 1 unit, then the morbidity rate will increase by 0.0016%. Because the population density in Medan City in 2020 is 9189.63, if the population density increases, the morbidity rate will also increase.

d. Assuming the predictors ($x_{1}$, $x_{2}$, $x_{3}$, $x_{5}$, $x_{6}$, $x_{7}$) are constant, then the effect of the open unemployment rates $\left(x_{4}\right)$ on the morbidity rate in 2020 (y) in Medan City can be written as follows.
  $${\hat{y}}_{30}=26.111x_{4;30}-26.463{\left(x_{4;30}-1.1514\right)}_{+}$$
  (38) $$\;=\left\{ \begin{array}{cc} 26.111x_{4;30} & ;x_{4;30}<1.1514\\ -0.352x_{4;30}+30.469 & ;x_{4;30}\geq 1.1514 \end{array}\right.$$

Based on the model obtained, it can be interpreted that if the open unemployment rate is less than 1.1514, it increases by 1%, and then the morbidity rate increases by 26.111%. Meanwhile, if the open unemployment rate is more than or equal to 1.1514, it increases by 1%, and the morbidity rate will decrease by 0.352%. Because the open unemployment rate in Medan City in 2020 is 10.74, if the open unemployment rate increases, the morbidity rate will decrease.

e. Assuming the predictors ($x_{1}$, $x_{2}$, $x_{3}$, $x_{4}$, $x_{6}$, $x_{7}$) are constant, the effect of the number of public hospitals $\left(x_{5}\right)$ on the morbidity rate in 2020 (y) in Medan City can be written as follows.
  $${\hat{y}}_{30}=8.749x_{5;30}-8.868{\left(x_{5;30}-1.34\right)}_{+}$$
  (39) $$\;=\left\{ \begin{array}{cc} 8.749x_{5;30} & ;x_{5;30}<1.34\\ -0.119x_{5;30}-11.884 & ;x_{5;30}\geq 1.34 \end{array}\right.$$

Based on the model obtained, it can be interpreted that if the number of public hospitals is less than 1.34 then it increases by 1 unit, then the morbidity rate will increase by 8,749%. Meanwhile, if the number of public hospitals is more than or equal to 1.34 then it increases by 1 unit, then the morbidity rate will decrease by 0.119%. Because the number of public hospitals in Medan City in 2020 is 67, if the number of public hospitals experiences more units, the morbidity rate will decrease.

f. Assuming the predictors ($x_{1}$, $x_{2}$, $x_{3}$, $x_{4}$, $x_{5}$, $x_{7}$) are constant, then the effect of the percentage of households that have access to safe drinking water$\;(x_{6})$ on the morbidity rate in 2020 (y) in Medan City can be written as follows.

${\hat{y}}_{30}=2.092x_{6;30}-1.964{\left(x_{6;30}-44.4184\right)}_{+}$
$\;=\left\{ \begin{array}{cc} 2.092x_{6;30} & ;x_{6;30}<44.4184\\ 0.128x_{6;30}+87.239 & ;x_{6;30}\geq 44.4184 \end{array}\right.\;$(40)

Based on the model obtained, it can be interpreted that if the percentage of households that have access to safe drinking water is less than 44,4184%, then it will increase by 1%, and then the morbidity rate will increase by 2,092%. Meanwhile, if the percentage of households that have access to proper drinking water is more than or equal to 44,4184 then it increases by 1%, then the morbidity rate will increase by 0.128%. Because the percentage of households that have access to safe drinking water in Medan City in 2020 is 98.79, if the percentage of households that have access to safe drinking water increases, the morbidity rate will increase.

g. Assuming the predictors ($x_{1}$, $x_{2}$, $x_{3}$, $x_{4}$, $x_{5}$, $x_{6}$) are constant, then the effect of the length of schooling $\left(x_{7}\right)$ on the morbidity rate in 2020 (y) in Medan City can be written as follows.
  $${\hat{y}}_{30}=-1.853x_{7;30}-2.354{\left(x_{7;30}-5.4806\right)}_{+}$$
  (41) $$\;=\left\{ \begin{array}{cc} -1.853x_{7;30} & ;x_{7;30}<5.4806\\ -4.354x_{7;30}+12.901 & ;x_{7;30}\geq 5.4806 \end{array}\right.$$

Based on the model obtained, it can be interpreted that if the average length of schooling is less than 5.4806 years, then it increases for one year, and then the morbidity rate will decrease by 1.853%. Meanwhile, if the average length of schooling is more than or equal to 5,4806 the next year, it increases for one year, then the morbidity rate will decrease by 4,354%. Because the average length of schooling in Medan City in 2020 is 11.39, if the average length of schooling increases, the morbidity rate will decrease.

## Model comparison

Based on the modeling, the level of goodness of the model is obtained based on the coefficient of determination from the GWTSNR model with the TSNR as the global regression as follows. Based on data analysis, it is obtained that the GWTSNR model with a one-knot point has a determination coefficient adjusted r-square of 96.235%, which is greater than the coefficient of determination (adjusted r-square) from TSNR model with a one-knot point is 70.159%. This indicates that the GWTSNR model with a one-knot point is the best model by being able to explain the effect of the predictor variables $x_{1},x_{2},x_{3},x_{4},x_{5},x_{6},$ and $x_{7}$ to the morbidity rate variable y of 96.235%.

## Conclusion

Morbidity rate data in North Sumatra 2020 has regression curves between predictor variables and the response variable does not determine a certain pattern. And morbidity rate in North Sumatra 2020 also has a spatial effect. There are eight regional groupings based on significant predictors with different effects on each group. Modeling the morbidity rate using the GWTSNR model with one-knot point has a coefficient of determination (adjusted r-square) of 96.235% which is greater than the coefficient of determination (adjusted r-square) of TSNR with one-knot point of 70.159%. This indicates that the GWTSNR model with a one-knot point is the best model by being able to explain the effect of the predictor variables$\;x_{1}$, $x_{2}$, $x_{3}$, $x_{4}$, $x_{5}$, $x_{6}\;$and $x_{7}$ on the morbidity rate of 96.235%.

## Ethics statements

The data used in this study is the morbidity rate data in North Sumatra in 2020. The data is secondary data accessed from the official website of the North Sumatra Provincial Health Office (https://bit.ly/3a3g2Xw) in the publication of North Sumatra Provincial Health Office Performance Report in 2020. The predictor variables used in this study are secondary data accessed on the website of the Central Statistics Agency of North Sumatra (https://sumut.bps.go.id/), or there are in the publication of BPS North Sumatra with the title North Sumatra Province in Figures 2021.

## CRediT author statement

Sifriyani, Gunardi, S.H. Kartiko, dan I. N. Budiantara: Methodology

Gunardi: Conceptual

Gunardi, Herni Utami, Zulaela, Sumardi: Writing-Reviewing

## Declaration of Competing Interest

The authors declare that they have no known competing financial interests or personal relationships that could have appeared to influence the work reported in this paper.

## Data Availability

Data will be made available on request.
